# The Relationship Between Hemoglobin A1c, Time in Range, and Glycemic Management Indicator in Patients With Type 1 and Type 2 Diabetes in a Tertiary Care Hospital in Saudi Arabia

**DOI:** 10.7759/cureus.63947

**Published:** 2024-07-06

**Authors:** Ahmed A Alazmi, Imad Brema, Saad H Alzahrani, Mohammed S Almehthel

**Affiliations:** 1 Obesity, Endocrine, and Metabolism Center, King Fahad Medical City, Riyadh, SAU; 2 Family Medicine and Endocrine Department, King Fahad Specialist Hospital, Dammam, SAU; 3 College of Medicine, Alfaisal University, Riyadh, SAU; 4 Division of Endocrinology, University of British Columbia, Vancouver, CAN

**Keywords:** hypoglycemia, flash glucose monitoring, glycemia management indicator, time in range, diabetes

## Abstract

Objectives: This study aimed to assess the correlation between glycated hemoglobin A1 (HbA1c), time in range (TIR), and glycemic management indicator (GMI) in patients with both type 1 diabetes (T1D) and type 2 diabetes (T2D) who were using a flash glucose monitoring (FGM) device (FreeStyle Libre; Abbott Diabetic Care, Witney, UK).

Methods: This was a retrospective study that looked at T1D and T2D FreeStyle Libre users' LibreView database in the period between January 2020 to June 2022. The study was conducted at the diabetes department at the King Fahad Medical City (KFMC) in Riyadh, Saudi Arabia, following Institutional Review Board (IRB) approval. Data were collected from the LibreView website, as well as from the electronic privacy information center (EPIC) hospital records.

Results: Data were available for 327 patients, mean age of 33.08(±17.1) years old, and 55.7% were females. HbA1c had a statistically significant correlation with both TIR and GMI with coefficient of correlation (r) values of 0.78 (p<0.001) and 0.82 (p<0.001), respectively. A linear regression model between TIR and Hb1Ac was also developed and found to be statistically significant (p<0.001) with an acceptable R^2^ value (0.60).

Conclusion: Study findings revealed that the %TIR could be a reliable predictor of Hb1Ac. Thus, Freestyle Libre was able to determine Hb1Ac as close to the lab results as possible. Therefore, it is necessary to encourage diabetes patients to achieve at least 70% TIR in order to keep Hb1Ac within the desired range.

## Introduction

For many years, glycated hemoglobin (HbA1c) has been used as the gold standard to assess the glycemic control of subjects with diabetes, and it has correlated well with the mean blood glucose levels, as well as with the risk of developing microvascular and macrovascular complications [1,2]. However, HbA1c has its own limitations, such as unreliability in cases of altered red blood cell lifespan, hemoglobinopathies, end-stage renal disease, and the fact that it takes at least two to three months to show changes in glycemic status [3-7]. Another limitation of A1C is that it can only reflect hyperglycemia without providing additional information about hypoglycemia, hyperglycemia, or glycemic variability [8].

To counter the limitations of Hb1Ac, continuous glucose monitoring (CGM) devices have been developed to monitor blood glucose levels throughout the day and night continuously. The use of these devices has increased dramatically over the past decade [9,10]. A person can check their blood glucose level at a glance, and it helps review how their blood glucose level has changed over the last few hours, allowing them to adjust their food intake, physical activity, and medications accordingly [11]. CGM can help reduce hypoglycaemic events and improve HbA1c levels and quality of life [12].

FreeStyle Libre (Abbott Diabetic Care, Witney, UK) is a flash glucose monitoring system, often referred to as flash CGM, that has been approved by the Food and Drugs Administration (FDA) to monitor glucose in people with diabetes [13]. The flash glucose monitoring (FGM) device (FreeStyle Libre) mechanically reads and continuously measures the glucose concentration in the interstitial fluid glucose collected from the cells immediately below the skin and produces the corresponding ambulatory glucose profile (AGP) by downloading the glucose measurements from the sensor to the reader [14]. Large, randomized control trials provided evidence that the use of flash CGM devices has resulted in significant reductions in hypoglycemia rates, increased time in the target range, reduced glycemic variability, and greater rates of patient satisfaction [14-16]. In addition, there is evidence that high FGM device utilization has perhaps resulted in increased patient engagement in managing their diabetes [17].

There has been a very limited number of studies that were conducted in Saudi Arabia to evaluate the use, acceptability, and success of CGM devices among patients with either type 1 diabetes (T1D) or type 2 diabetes (T2D). In 2021, Al Hayek et al. reported their data on patients with T2D who are using FGM devices focusing on patients’ satisfaction among other outcomes achieved [18]. Another study was published in 2022 by Al-Harbi et. al and reported that higher scanning frequency was associated with a lower estimated A1c, higher time in range (TIR), lower glucose variability, and less time in hypoglycemia or hyperglycemia among FGM device users in Saudi Arabia [19]. Moreover, a limited number of studies have been conducted to evaluate the use of FGM devices in subjects with T2D or insulin pump users [20,21]. Nonetheless, the correlation between TIR and HbA1c has not been studied in Saudi patients with diabetes. Therefore, the aim of this study was to assess the correlation between HbA1c and TIR in patients with diabetes who are using FGM devices in Saudi Arabia.

## Materials and methods

This retrospective study was conducted at the diabetes department at the King Fahad Medical City (KFMC) in Riyadh, Saudi Arabia. The study was approved by the institutional review board (IRB) of the KFMC, with IRB log no. 22-327 (July 27, 2022). Data for the FreeStyle Libre was taken from the “LibreVeiw” website, and demographic data were taken from the hospital records.

The inclusion criteria were as follows: (1) subjects should be aged 14 years and above; (2) subjects must have either type 1 or type 2 diabetes only; and (3) subjects must use the FreeStyle Libre glucose monitoring system for at least a 90-day duration with at least 70% active sensor time. The exclusion criteria were as follows: (1) subjects under 14 years of age; (2) subjects with other types of diabetes that are not specified above; and (3) subjects who used FreeStyle Libre for less than 90 days and/or used the sensor for less than 70% of the time.

The variables collected from the “LibreVeiw” website were glycemic management indicator (GMI, %), TIR (%; time glucose levels were between 70 and 180 mg/dL), time above range (TAR, %; time glucose levels were >180 mg/dL and <250 mg/dL), time below range (TBR, %; time glucose levels were <70 mg/dL (American Diabetes Association "ADA" level 1 hypoglycemia) and <54 mg/dL (ADA level 2 hypoglycemia), and coefficient of variation (CoV, %). Variables that were taken from hospital records were age, gender, type of diabetes, and lab HbA1c. The recent Hb1Ac test result was obtained from the hospital records, and 90 days of data was taken from the LibreVeiw website. The 90-day analysis period included the 90 days preceding the day at which the blood sample for HbA1c was taken.

Statistical analysis

The data were analyzed using Statistical Product and Service Solutions (SPSS, v. 23; IBM SPSS Statistics for Windows, Armonk, NY). Descriptive analysis of the data included averages, standard deviation (SD), percentages, and graphical data presentation. The normality of the data was tested by using the Shapiro-Wilk test, which revealed that the data were not normally distributed. Hence, non-parametric tests were performed in the inferential data analysis. The Mann-Whitney U test was performed to study the variation in averages due to the variables having two categories. The correlation between HbA1c and %TIR and between HbA1c and GMI was calculated by using Spearman rank correlation. The linear regression model was constructed by using HbA1c as the dependent and %TIR as the independent variable. All p-values less than 0.05 were considered statistically significant.

## Results

The total number of participants was 327. A total of 182/327 of the study subjects (55.7%) were females. About 252/327 of the subjects (77.8%) had T1D, while 72 (22.2%) had T2D. Table 1 shows the baseline characteristics of the study population. The average age of participants was 33.08 (±17.1) years, average TIR (70-180 mg/dL) achieved was 52.72%, average TAR (>180 mg/dL) was 38.86%, average TBR (>70 mg/dL) was 5%, and average glucose variability (assessed by CV%) was 39.79%. Average GMI% and HbA1c% were 7.57% and 7.86% respectively with an average sensor usage time of 86.47%.

**Table 1:** Baseline characteristics of the study population TIR, time in range; TBR, time below range; TAR, time above range; GMI, glycemic management indicator, CoV, coefficient of variation; HbA1c, glycated hemoglobin A1c

Variable	Mean (SD)
Age (years)	33.08 (17.1)
Active Sensor Time (%)	86.47 (10.3)
TIR (%)	52.72 (17.7)
TBR (%)	5.0 (5.3)
TAR (%)	38.86 (7.9)
GMI (%)	7.57 (0.9)
CoV (%)	39.79 (18.1)
Hb1Ac (%)	7.86 (1.2)

Differences in averages in the variables between male and female subjects and between subjects with T1D and subjects with T2D are presented in Table 2. Female subjects had higher TBR (p=0.037) and lower TIR (p=0.044). The coefficient of variation (CoV) was higher among female subjects as well (p=0.004).

**Table 2:** Association between gender and type of diabetes and scale variables TIR, time in range; TBR, time below range; TAR, time above range; GMI, glycemic management indicator, CoV, coefficient of variation; HbA1c, glycated hemoglobin A1c *Statistically significance at p < 0.05

Variable	Mean (SD)		P-value	Mean (SD)		P-value
	Male	Female		T1DM	T2DM	
TBR (%)	4.3 (4.7)	5.55 (5.7)	0.037^*^	5.87 (5.5)	2.06 (3.1)	0.000^*^
TAR (%)	40.78 (19.1)	43.43 (19.3)	0.121	44.15 (18.2)	35.25 (21.3)	0.000^*^
CoV (%)	37.66 (7.6)	39.81 (8.0)	0.004^*^	40.98 (7.2)	31.41 (5.7)	0.000^*^
GMI (%)	7.52 (0.9)	7.61 (0.9)	0.113	7.63 (0.9)	7.34 (0.9)	0.003^*^
Hb1Ac (%)	7.73 (1.2)	7.97 (1.3)	0.062	7.86 (1.25)	7.85 (1.24)	0.740
TIR (%)	54.86 (18.1)	51.03 (17.3)	0.044^*^	49.94 (15.8)	62.69 (20.6)	0.000^*^
Active Sensor Time (%)	87.19 (10.2)	85.9 (10.3)	0.227	85.69 (10.3)	89.13 (10.0)	0.007^*^
Average Glucose	176.2 (37.6)	179.7 (38.6)	0.132	180.59 (37.5)	169.18 (39.9)	0.0031^*^

Subjects with T2D had lower TBR (p<0.001), while TAR was higher in subjects with T2D (p<0.001). Additionally, CoV was higher among subjects with T1D (p<0.001). GMI and average glucose levels were higher in subjects with T1D (p-values=0.003 and 0.0031, respectively). On the other hand, TIR and active sensor time were higher among subjects with T2D (p-values=0.000 and 0.007, respectively).

**Figure 1:**
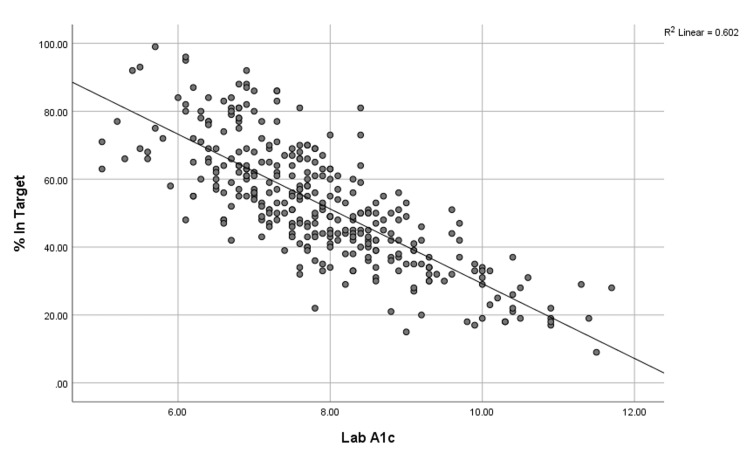
Relationship between Hb1Ac and %TIR HbA1c, glycated hemoglobin A1; TIR, time in range

**Figure 2:**
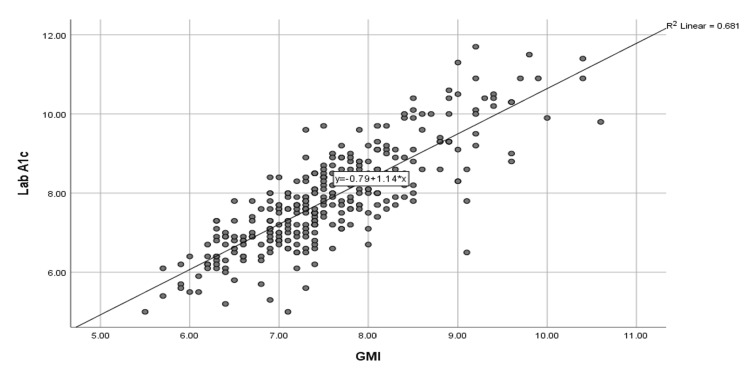
Relationship between Hb1Ac and GMI Dependent variable: Lab A1c, * Statistically significant at 0.05 level of significance HbA1c, glycated hemoglobin A1c; GMI, glycemic management indicator

Figure 1 shows the inverse correlation between Hb1Ac and %TIR. The correlation was strong, inverse, and statistically significant (r=-0.776; p=0.000), while the correlation between Hb1Ac and GMI was strong, direct, and statistically significant (r=0.825; p=0.000) (Figure 2). In addition, a positive weak but statistically significant correlation was found between the CoV and Hb1Ac (r=0.264; p=0.025) among subjects with T2D. Therefore, the linear regression model was derived in which Hb1Ac was used as a dependent variable, while %TIR was used as the independent variable. The regression model was found statistically significant (p<0.001) with a high R2 value (0.60), and the error was 0.79, which also showed the accuracy of the regression model. Table 3 presents the constant and slopes for the regression model with p-values. Table 4 reveals the estimated values of A1C for the given values of %TIR.

**Table 3:** Coefficients for the multiple linear regression model * Statistically significant at the 0.05 level of significance

Model	Slope	Std. Error	t-value	p-value
(Constant)	10.75	0.137	78.36	0.000^*^
% In Target	-0.055	0.002	-22.19	0.000^*^

**Table 4:** Estimate of A1c for a given TIR level

TIR 70-180 mg/dL	A1c, % (mmol/mol)
20%	9.65
30%	9.1
40%	8.55
50%	8
60%	7.45
70%	6.9
80%	6.35
90%	5.8

## Discussion

The evidence from landmark trials, such as DCCT (Diabetes Chronic Complications Trial) and UKPDS (United Kingdom Prospective Diabetes Study), supports the use of HbA1c as a glycemic target for people with diabetes; however, it has many limitations, such as the limited ability to assess acute glucose fluctuations, mainly hypoglycemia and hyperglycemia as well as glucose variability [1,2]. Therefore, it is conceivable that Hb1Ac alone is inadequate in providing optimal information about the overall patients' glycemic control [22]. On the other hand, CGM provides a viable alternative to give more comprehensive information about glucose profiles, which is very important in managing patients with diabetes.

Many studies have been conducted to validate the collected data from continuous glucose monitoring devices and report their accuracy. However, there is a scarcity of evidence regarding the performance and accuracy of the Freestyle Libre System in the Saudi population, with very few exceptions [21]. Therefore, this study provided data on the accuracy of the results obtained from FreeStyle Libre on a Saudi population with both T1D and T2D.

In the present study, we report a significant correlation between Hb1Ac and TIR, which is consistent with previously published studies [23,24]. In their study, Vigersky et al. reported a strong correlation between Hb1Ac and TIR, which included both T1DM and T2DM patients [23). Moreover, Beck et al. investigated the same relationship between the two metrics in their clinical trial, which included 545 patients with T1D, and they described a significant linear correlation between the TIR and lab HbA1c [24). In the two previous studies, the correlation between TIR and Hb1Ac reported a coefficient of correlation -0.85 and -0.67, respectively [23,24]. 

According to the international consensus on TIR, TIR should be >70% in most patients with T1D and T2D [25]. Our patients with T2D achieved a significantly higher TIR (62.69%±20.6) than those with T1D (49.94%±15.9). Our findings are consistent with the study conducted by Cutruzzola et al. in 2020, which reported that patients with T2D had a significantly higher percentage of points in range (PIR) than patients with T1D [26]. Most of the published studies included either subjects with T1D only or T2D only, and very limited studies included patients with both types of diabetes [22,27,28]. An interesting finding that we found in our study is that male subjects achieved a significantly higher TIR than female subjects, with significantly less variation.

We have also found a very strong inverse correlation between TIR and HbA1c in subjects with T1D with a very good coefficient of correlation of 0.77, compared to the previous studies, which reported a lower degree of correlation with a coefficient of correlation between 0.69 and 0.53, respectively, between these two variables [29,30].

The present study reports a positive correlation between HbA1c and CoV among subjects with T1D and T2D, which is an important finding to describe despite the less strong degree of correlation because CoV could be an independent predictor of diabetes complications, as described in some studies [31-33]. TIR has been shown to be a possible determinant of the future risk of diabetic complications in several studies, both in T1D and T2D [34-36].

The main limitations of the current study are the relatively small sample size, the retrospective design, and the fact that it was a single-center study, which may hamper the study’s generalizability. Thus, a multicenter study with a larger sample size is warranted to support our findings.

## Conclusions

In conclusion, we report a strong and significant inverse relationship between TIR and laboratory-measured HbA1c in a large cohort of Saudi patients with T1D and T2D. In addition, we report a strong linear correlation between the GMI and laboratory-measured HbA1c. Based on our data and other data, it would be reasonable to propose the use of Freestyle Libre metrics, such as GMI and TIR, in the decision-making process in some patients in situations where laboratory-measured HbA1c is not available or unreliable. However, there is a need to conduct large RCTs to look at the association between these metrics and long-term diabetes complications.
